# Muscle Fatigue Post-stroke Elicited From Kilohertz-Frequency Subthreshold Nerve Stimulation

**DOI:** 10.3389/fneur.2018.01061

**Published:** 2018-12-04

**Authors:** Yang Zheng, Henry Shin, Xiaogang Hu

**Affiliations:** Joint Department of Biomedical Engineering, University of North Carolina at Chapel Hill and North Carolina State University, Raleigh, NC, United States

**Keywords:** transcutaneous electrical nerve stimulation, electromyography, muscle fatigue, stroke, kilohertz-frequency

## Abstract

**Purpose:** Rapid muscle fatigue limits clinical applications of functional electrical stimulation (FES) for individuals with motor impairments. This study aimed to characterize the sustainability of muscle force elicited with a novel transcutaneous nerve stimulation technique.

**Method:** A hemiplegic chronic stroke survivor was recruited in this case study. Clustered subthreshold pulses of 60-μs with kilohertz (12.5 kHz) carrier frequency (high-frequency mode, HF) were delivered transcutaneously to the proximal segment of the median/ulnar nerve bundles to evaluate the finger flexor muscle fatigue on both sides of the stroke survivor. Conventional nerve stimulation technique with 600-μs pulses at 30 Hz (low-frequency mode, LF) served as the control condition. Fatigue was evoked by intermittently delivering 3-s stimulation trains with 1-s resting. For fair comparison, initial contraction forces (approximately 30% of the maximal voluntary contraction) were matched between the HF and LF modes. Muscle fatigue was evaluated through elicited finger flexion forces (amplitude and fluctuation) and muscle activation patterns quantified by high-density electromyography (EMG).

**Result:** Compared with those from the LF stimuli, the elicited forces declined more slowly, and the force plateau was higher under the HF stimulation for both the affected and contralateral sides, resulting in a more sustainable force output at higher levels. Meanwhile, the force fluctuation under the HF stimulation increased more slowly, and, thus, was smaller after successive stimulation trains compared with the LF stimuli, indicating a less synchronized activation of muscle fibers. The efficiency of the muscle activation, measured as the force-EMG ratio, was also higher in the HF stimulation mode.

**Conclusion:** Our results indicated that the HF nerve stimulation technique can reduce muscle fatigue in stroke survivors by maintaining a higher efficiency of muscle activations compared with the LF stimulation. The technique can help improve the performance of neurorehabilitation methods based on electrical stimulation, and facilitate the utility of FES in clinical populations.

## Introduction

Functional electrical stimulation (FES) can electrically activate muscle fibers with electrodes placed at the muscle belly, and can be used to promote functional improvement in paralyzed limbs due to neurological disorders (1, 2). Even though FES has shown some success in assisting individuals with neurological disorders, the clinical impact is hampered by a critical limitation which involves rapid muscle fatigue onset during repeated stimulations (3, 4). The increased muscle fatigability can arise from factors including the violation of the size principle of motor unit (MU) recruitment (5, 6) and the highly synchronized activation of muscle fibers. Furthermore, muscles from the paralyzed limbs are more fatigable compared with the muscles of the contralateral side or intact individuals due to central (7) or peripheral fatigue (8–10), which can further limit clinical applications of FES.

Previous studies have explored various techniques to reduce muscle fatigue. For example, prolonged force production has been observed by alternately stimulating muscle synergists (11) or using randomly modulated stimulation parameters to activate different muscle fibers separately (12–14). Similarly, multi-electrode stimulation techniques have been used to asynchronously activate different muscle portions (15–18). More recently, stimulation at the proximal segment of nerve bundles has been investigated (19, 20), in order to recruit a range of MUs, especially the more fatigue-resistant MUs. The nerve stimulation technique can result in less fatigable contractions (21) and more dexterous movements (22). In addition, the nerve bundle stimulation technique can activate afferent fibers with a low current amplitude and a high frequency (23), resulting in a more physiological recruitment order of MUs. However, the activations of different MUs are still highly synchronized, and the H-reflex activity is still secondary compared with the M-wave when large muscle forces are required.

In a previous study (24), clustered narrow pulses with a carrier frequency of kilohertz (high frequency, HF) targeting the proximal nerve bundles can lead to asynchronized activation of muscle fibers and reduced force fluctuations, compared with wide pulses delivered at a low frequency (LF). Furthermore, the HF stimulation technique can reduce muscle fatigue in healthy subjects (25). However, it is not known to what degree the HF stimulation technique can improve the force sustainability in paralyzed limbs of individuals with neurological disorders. Accordingly, a hemiplegic stroke survivor was recruited in the current case study. The elicited finger flexion forces and the EMG activity from the finger flexor muscles were compared between the HF and LF stimulation on both the affected (paretic) and contralateral sides.

## Methods

### Experiment and Subject

#### Subject Background

A 54-year-old chronic stroke survivor with severe motor impairment in the arm and hand was recruited. The subject suffered from an ischemic stroke in the left corona radiate 2 years prior to the testing. The Chedoke-McMaster Stroke Assessment of the hand component was 2, and the arm component was 3. No cognition impairment was reported. Two experimental sessions, with one on each side, were performed with an interval of 1 month. This study was carried out in accordance with the recommendations of the Institutional Review Board (IRB) of the University of North Carolina at Chapel Hill with written informed consent from the subject. The subject gave written informed consent in accordance with the Declaration of Helsinki. The protocol was approved by the local IRB. Additional written informed consent was obtained from the subject for the publication of this case report.

#### Experiment Setup

**Figure 1:**
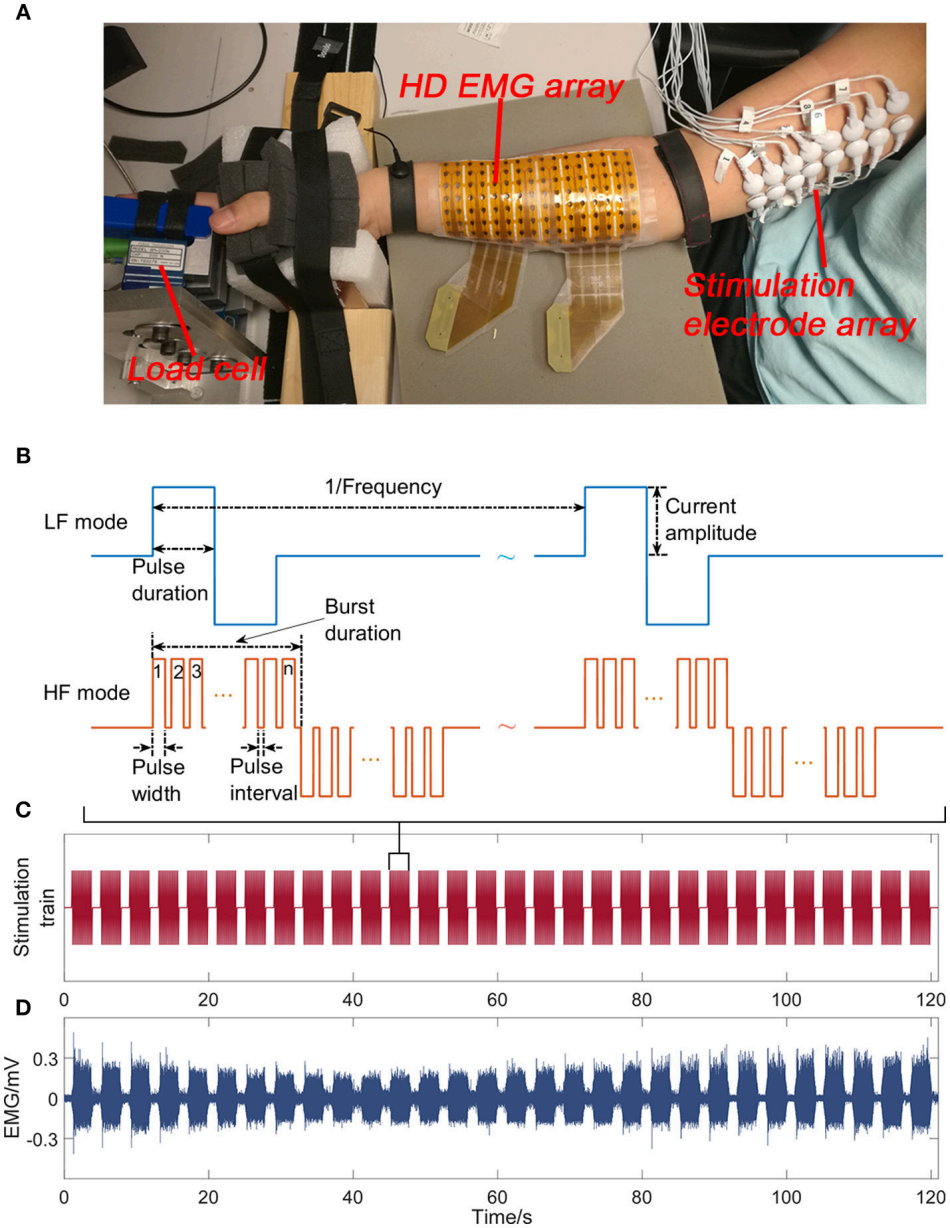
The experimental apparatus includes the stimulation of the ulnar/median nerve bundles, and the recordings of high density EMG at the forearm flexor muscles and the finger flexion forces **(A)**. Sample stimulation trains for the LF and HF stimulation mode **(B)**. The stimulation train of a trial on the contralateral side in the HF mode **(C)**. The EMG signal of a representative channel recorded on the contralateral side in the HF mode **(D)**.

The subject sat in an arm chair at a comfortable height with his back supported during the experiment. The hand was restrained, and the forearm was supported (Figure 1A). The individual fingers were secured to four miniature load cells (SM-200N, Interface), respectively for force measurements with a sample frequency of 1 kHz.

An 8 × 16 channel high density EMG electrode pad (OT Bioelettronica) was placed at the finger flexor muscles to obtain the EMG activities (Figure 1A). The electrode diameter was 3 mm with an inter-electrode distance of 10 mm. The center of the electrode grid was aligned with the midline between the olecranon process and the styloid process. Monopolar EMG signals were amplified using EMG-USB2+ (OT Bioelettronica). The sample rate, gain and bandwidth of the amplifier were set at 5,120, 200, and 10–900 Hz, respectively.

Sixteen (2 × 8 array) gel-based skin-surface stimulation electrodes (10 mm in diameter) were placed beneath the short head of the biceps brachii along the ulnar/median nerves. A custom-made MATLAB user interface was used to control a stimulator (STG4008, Multichannel Systems) and a switch matrix (Agilent Technologies) to deliver the stimulation trains with different parameters to any pair of the 16 electrodes.

#### Stimulation Paradigm

The sample stimulation trains for the LF and HF mode are illustrated in Figure 1B. In the LF mode, biphasic pulses were delivered at 30 Hz. In the HF mode, narrow pulses with a 60-μs pulse width were clustered in bursts with a 20-μs pulse interval, leading to a carrier frequency of 12.5 kHz. Different clusters were also delivered at 30 Hz. A pilot test demonstrated that a single 60 μs pulse evoked no EMG activity nor force responses.

The maximum voluntary contraction (MVC) of individual fingers was first obtained, when the subject maximally flexed the individual fingers against the load cell. Before the fatigue test, we first searched across all electrode pairs using the LF stimuli with a pulse duration of 600 μs to identify the electrode pair that can elicit the strongest muscle contraction with no pain sensation. Then, the current amplitude was adjusted until 30% of MVC was obtained for at least one finger. In the subsequent experiment, the current amplitude and the electrode pair were the same between the two stimulation modes. Muscle fatigue was evoked by delivering 20 (for the affected side) or 30 (for the contralateral side) 3-s stimulation trains with 1-s intervals (Figure 1C). Since muscles of the contralateral side are generally less fatigable than that of the affected side, additional stimulation trains were delivered to ensure that fatigue can be induced. Two trials with one stimulation mode in each trial were performed in a random order. The resulting trial order was that the LF mode was first tested on the affected side, and the HF mode was first tested on the contralateral side. The initial contraction forces from the two modes were matched such that 30% MVC was elicited in the same finger. A 10-min rest was provided between trials, which was sufficient for the subjects to recover from muscle fatigue with fully recovered forces under the stimulation protocol (26).

### Data Analysis

#### Finger Force

To compare the overall force output, the raw forces from all fingers were summated at individual sample points, and the force-time integral was calculated based on the summated force. To further quantify the decline of forces between the two modes, the raw force data from each 3-s stimulation train were first averaged for individual fingers. Then, the average forces from all fingers were summated to represent the contraction force level of individual stimulation trains. Next, the force level were fitted with an exponential function, i.e.*y* = *a* + *be*^τ*t*^, where *a* represents the force plateau level, *b* is the absolute force decay, and τ < 0 reflects the rate of force decline. A direct comparison of the τ value cannot accurately reflect the force decline rate because the force plateau under two different modes can be different. Therefore, we estimated the number of stimulation trains (termed as the 50%-peak stimulation) for the force to decline below 50% of the initial force level. A larger 50%-peak stimulation represents a slower force decay rate.

To estimate the force variability, the forces were first band-pass filtered (20–40 Hz) to eliminate the low-frequency offset and the high-frequency noise. Then, the standard deviation of the filtered force was calculated to represent the magnitude of the force fluctuation for individual stimulation trains. As in our previous study (25), the summated force fluctuation across all fingers was further normalized by the absolute force level of individual stimulation trains to eliminate the effect of force decline.

#### EMG Activity

Within each 3-s stimulation train, the EMG segments 5 ms prior to and 35 ms after the stimulation onset were averaged to obtain the average EMG for individual channels. Stimulation artifacts were then identified with a threshold-crossing method based on the EMG, and the EMG segment between two consecutive stimulation artifacts were extracted to calculate the EMG area, estimated as the integral of the absolute EMG over time (Figure 1D). The start and end point of the EMG integral calculation remained the same across the two stimulation modes. The estimated EMG areas were then averaged across all 128 channels to represent the overall level of the EMG activity. To evaluate the efficiency of muscle force generation, the force-to-EMG area ratio (F–EMG ratio) was calculated.

### Results

**Figure 2:**
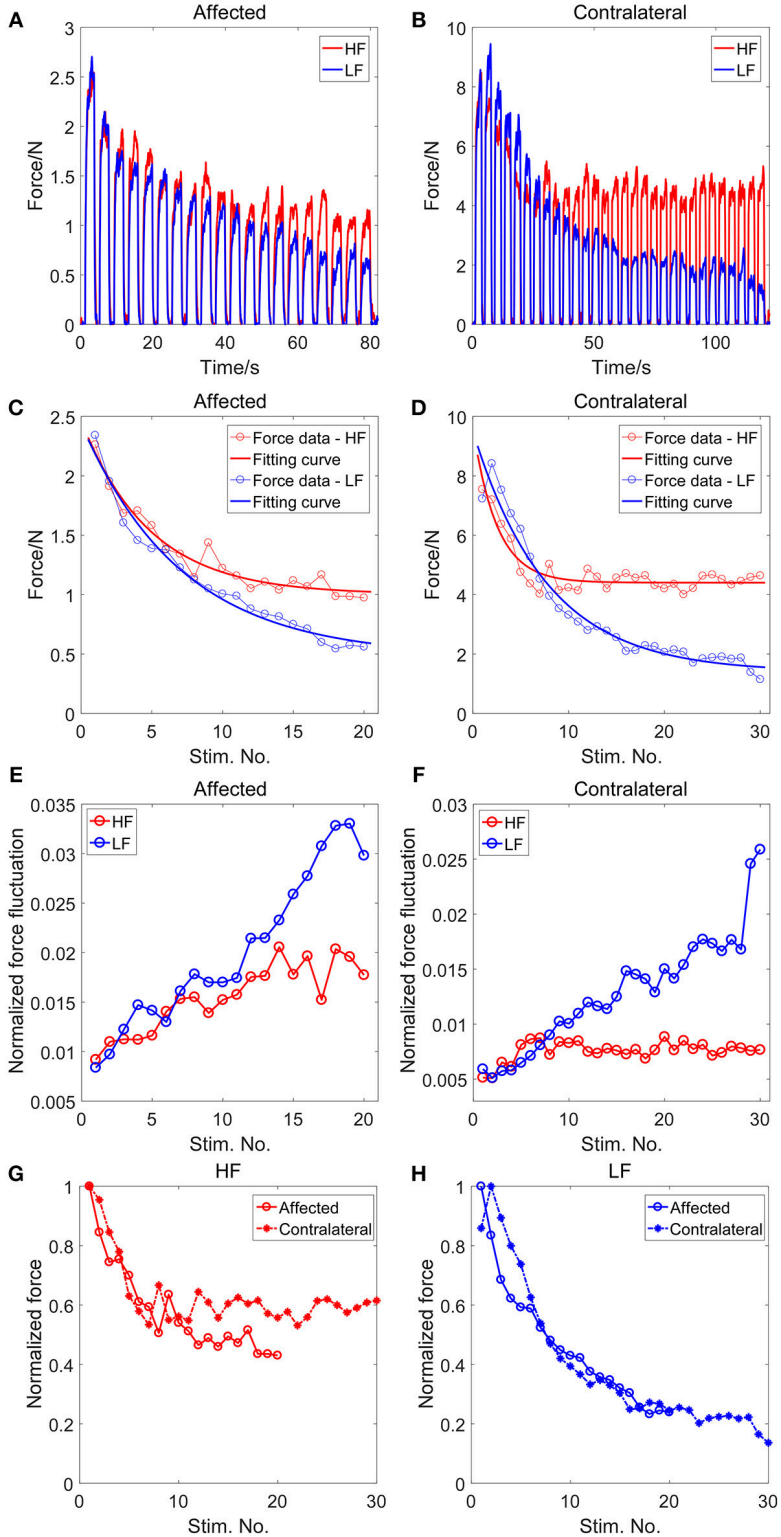
The summated force across fingers for the affected **(A)** and the contralateral **(B)** side. The changes of the force level with the sequential stimulation trains for the affected **(C)** and the contralateral **(D)** side, and the corresponding fitting results with the exponential function. The changes of the normalized force fluctuation with the successive stimulation trains for the affected **(E)** and the contralateral **(F)** side. Comparison of the normalized force between the affected and contralateral sides under the HF stimulaion **(G)** and the LF stimulaion **(H)**.

Figures 2A,B illustrate the summated force of all four fingers between the two stimulation modes for the affected and contralateral sides. Each impulsive force was elicited by a stimulation train, resulting in 20 and 30 force pulses for the affected and contralateral sides, respectively. With matched initial force levels, the force declined notably as successive LF stimulation trains were delivered. In contrast, the force declined more slowly and stayed at a higher level under the HF stimulation. Compared with the LF mode, the force-time integral under the HF stimulation was 24.38 and 45.63% larger in the affected and contralateral side, respectively, indicating more force outputs under the HF stimulation (Table 1).

**Table 1:** Comparison of fatigue measurements.

	**Affected**		**Contralateral**	
	**HF**	**LF**	**HF**	**LF**
Force-time integral/Ns	68.83	55.34	367.06	252.05
Force plateau/N	1.00	0.48	4.40	1.40
Force decay/N	1.46	1.96	5.26	8.12
50%-peak stimulation	14	9	>30	9

The changes of the contraction force are illustrated in Figures 2C,D for the affected and contralateral sides, respectively. For all conditions, the force decreased exponentially with the stimulation train. The average R-squared value of the exponential fit across all conditions was 0.9432 ± 0.0386. For both the affected and contralateral sides, the force decay was smaller and the force plateau was higher after successive HF stimulation trains, compared with that in the LF stimulation (Table 1). Under the LF stimuli, only 9 stimulation trains were needed for the force to decline below 50% of the initial force level for both the affected and contralateral sides. On the contrary, the 50%-peak stimulation under the HF stimuli was 14 for the affected side and was even larger than 30 for the contralateral side (Table 1). Figures 2G,H illustrate the relative force decay between the affected and contralateral sides under different stimulation modes. Under the HF stimulation, the force had a higher plateau on the contralateral side compared with the affected side. Under the LF stimulation, on the contrary, the force declined continuously for both sides and the decay rate was similar between the two sides.

The normalized force fluctuation increased with successive stimulation trains (Figures 2E,F), which was consistent with our previous study (25). The increase of the normalized force fluctuation was larger under the LF stimulation compared with that under the HF stimulation for both the affected and contralateral sides.

**Figure 3:**
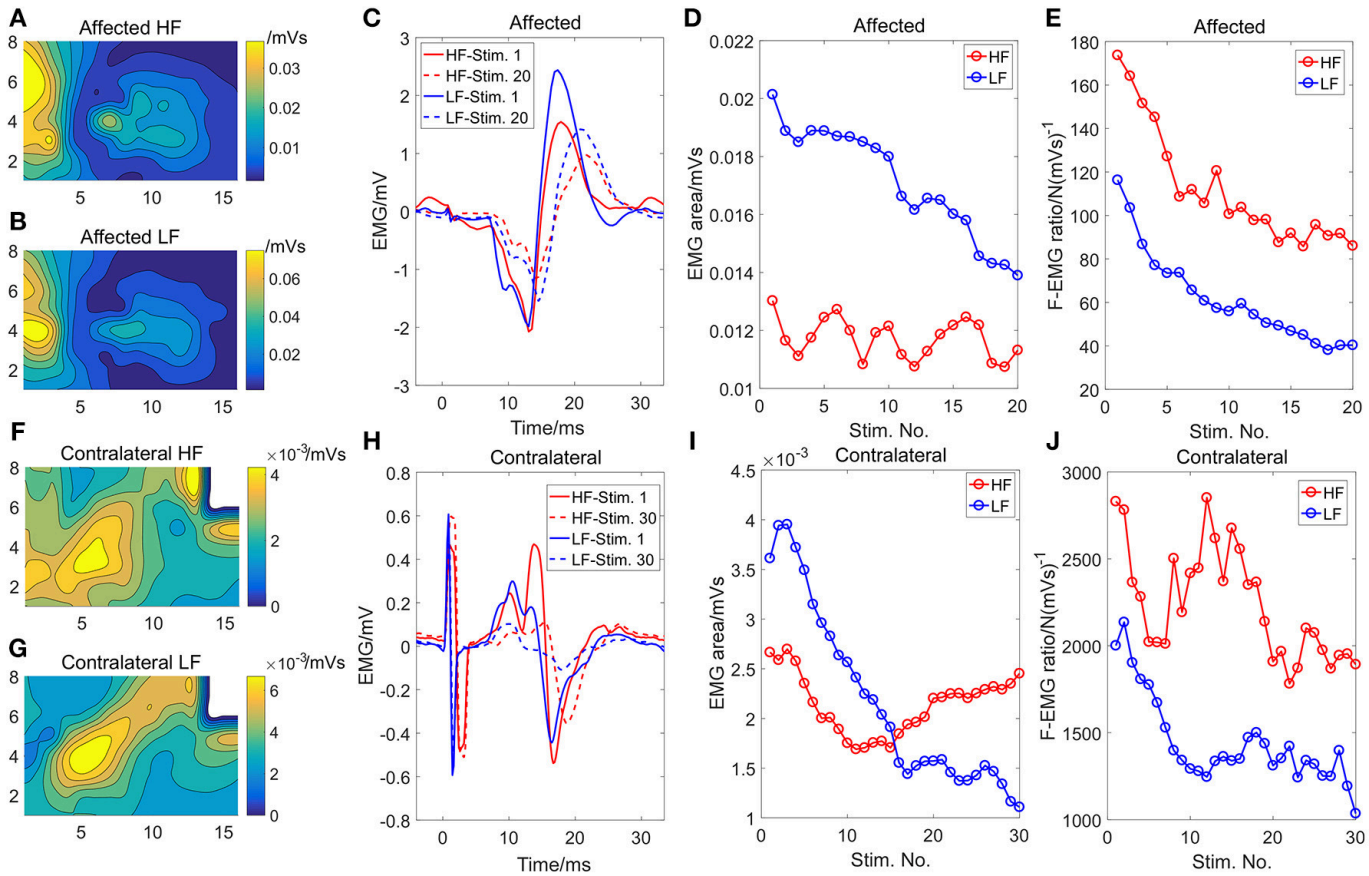
The initial (first stimulation train) strength distribution of the EMG activity under the HF **(A)** and the LF **(B)** stimulation, respectively, on the affected side. The initial strength distribution of the EMG activity under the HF **(F)** and the LF **(G)** stimulation, respectively, on the contralateral side. The white area in **(F)** and **(G)** was eliminated from analysis because of motion artifact contamination. The EMG activity of the channel with the maximum EMG area on the affected **(C)** and the contralateral sides **(H)**, respectively. Only the EMG activity of the first and the last stimulation train were illustrated. The changes of the EMG area with the successive stimulation trains for the affected **(D)** and contralateral sides **(I)**, respectively. The changes of the F-EMG ratio with the successive stimulation trains for the affected **(E)** and the contralateral sides **(J)**, respectively.

The initial EMG activity distribution and the average EMG of the channel with the highest intensity from the first and the last 3-s stimulation train are shown in Figure 3. Similar contour lines between the two modes demonstrated that similar muscles or muscle portions were activated between the two modes for both sides. The amplitude of the EMG activity under the HF stimulation was smaller compared with the LF mode. The EMG activity intensity decreased substantially over time under the LF stimulation (Figures 3D,I) while the EMG activity under the HF stimulation did not show an obvious declining trend. The F-EMG ratio decreased in both stimulation modes, indicating a decreased efficiency of the muscle force generation after fatigue. However, the F-EMG ratio under the HF stimulation was consistently larger than that under the LF stimulation over time, indicating a consistently higher efficiency of force generation under the HF stimulation.

## Discussions

In this case study, a HF stimulation protocol was tested on a hemiplegic stroke survivor to verify whether it can reduce muscle fatigue compared with the conventional LF stimulation. The results showed that the elicited force declined more slowly and the force plateau was higher under the HF stimulation for both sides, resulting in a more sustainable force output at higher levels. The force fluctuation increased more slowly, and was smaller with successive HF stimulation trains compared with the LF stimuli, indicating a less synchronized activation of the muscle fibers. The efficiency of the muscle activation measured as the F-EMG ratio (27) was higher after successive HF stimulation trains. These results indicate that the HF stimulation has the potential to reduce muscle fatigue in stroke survivors, compared with the LF stimulation, by maintaining a higher efficiency of muscle activations.

The faster fatigue onset during electrical nerve stimulation can arise from the differences between physiologically activated MUs and electrically excited MUs. First, the recruitment order differs between the two activations (5, 6). FES preferentially excites the larger axons with lower resistance that innervate the more fatigable muscle fibers (28), although a random recruitment order has also been observed (29). Second, the electrical activation of MUs are highly synchronized and the twitch forces are time-locked to the stimulation. This is contrary to voluntary contraction where different MUs discharge asynchronously at a range of different firing rates. Therefore, the highly synchronized and physiologically reversed recruitment of MUs during FES replace the normal physiological recruitment of MUs and their discharge regulation, resulting in a fast decline of the elicited forces.

The mechanisms underlying reduced muscle fatigue in the HF stimulation may be multifactorial. First, the HF stimulation can temporally disperse the activation of different motor units (24). Each narrow pulse can only induce a subthreshold depolarization of the axon membrane and different axons might need different numbers of subthreshold depolarizations to reach the threshold, resulting in an enlarged delay between the onsets of action potentials of different axons. Since previous studies have demonstrated that asynchronized activations of different muscle fibers can delay the onset of muscle fatigue (15–17), the decreased synchronization level of motor units with the HF stimulation can thus be a possible mechanism for the higher fatigue-resistance. Second, the HF mode stimulation could elicit more H-reflex activities compared with the LF stimulation. At low levels of stimulation intensity, the Ia afferent fibers are preferentially activated due to their intrinsic properties and their larger diameters compared with the motor axons. Within each burst of the HF stimulation, the current was delivered intermittently. Since the Ia afferent fibers can be activated more easily than the motor axons, the HF stimulation burst could first activate Ia afferent fibers compared with the single wide pulse in the LF mode. As a result, a less number of action potentials may propagate antidromically along the motor axons, and more H-reflex activities (30) can be observed. H-reflex can lead to more physiological recruitment of the motor units. The initial EMG distribution showed similar patterns between the two stimulation modes, indicating similar muscles or muscle portions were activated. However, the H-reflex activity was still considered as a possible mechanism because the surface EMG grid can only capture the activities of superficial muscles and not the EMG activities of deep muscles through the H-reflex pathway. Lastly, a potentially changed axon excitability can also lead to reduced force generation (31). Compared with the LF mode, the HF mode possibly leads to fewer axon drop-out due to a smaller increment of the axon threshold associated with fatigue.

Even though the HF stimulation can prolong the force output for both the affected and contralateral sides, the amount of increased force outcome on the affected side was smaller compared with the contralateral side (Table 1 and Figure 2G). The possible reason is that the muscle fibers of the paralyzed limbs after neurological disorders are innately more fatigable than muscle fibers on the contralateral side (7–10). An additional piece of support for this lies in the major differences in the decline of F-EMG ratio between the two sides. Although the F-EMG ratio after successive HF stimulations plateaued in the contralateral limb, which is consistent with our previous study (25), the affected limb showed continuous decline of F-EMG ratio. This difference can arise from a relatively higher force level of the affected side, given that the subject was severely impaired. The other possible explanation is the disrupted muscle force transmission to the tendon of the affected muscles following stroke (32). The transmission failure of the muscle force to the tendon counteracts the influence of the HF stimulation on muscle fiber activation. Therefore, even though the affected muscle still showed a decrease in the rate of force decline with HF stimulation, the improvement of fatigue resistance was smaller compared with that on the contralateral side.

## Concluding Remarks

The current study showed that the HF stimulation at the proximal segment of the nerve bundles can reduce muscle fatigue in a stroke survivor compared with the conventional LF stimulation. The outcomes demonstrated the clinical significance of the technique to elicit sustainable muscle contractions at high force levels for individuals with neurological disorders.

## Author Contributions

YZ and XH conceptualized the study, YZ and HS performed the experiment, YZ analyzed the data, YZ and HS drafted the manuscript. YZ, HS, and XH revised the manuscript and approved the final version.

### Conflict of Interest Statement

The authors declare that the research was conducted in the absence of any commercial or financial relationships that could be construed as a potential conflict of interest.
